# Exploring the Interpersonal Goals of Autistic and Neurotypical Adolescents Who Bully Others

**DOI:** 10.1007/s10803-024-06683-x

**Published:** 2024-12-17

**Authors:** Elian Fink, Samantha Friedman, Tjeert Olthof, Sandra van der Meijden, Frits Goossens, Sander Begeer

**Affiliations:** 1 School of Psychology, Pevensey Building, University of Sussex, Falmer, BN1 9QH UK; 2 University of Edinburgh, Edinburgh, UK; 3 Vrije Universiteit Amsterdam, Amsterdam, Netherlands

**Keywords:** Autism, Bullying, Interpersonal goals, Adolescence, Specialist education provision

## Abstract

The current study examined the association between interpersonal social goals (i.e., agentic and communal goals) and bullying behaviour for autistic adolescents (*n* = 108, *M*_age_ = 15.25 years, *SD* = 1.65) and neurotypical adolescents (*n* = 592, *M*_*age*_ = 13 years, *SD* = 0.5). Bullying behaviour was assessed using both self- and peer-reported measures. Agentic and communal social goals were assessed using the child version of the Interpersonal Goal Index. Measurement properties of the Interpersonal Goal Index were first examined, and some features were found to differ across autistic and neurotypical adolescents. Bullying behaviour was associated with agentic goals for neurotypical adolescents whereas communal goals were associated with bullying for autistic adolescents, suggesting a mismatch between social goals and social behaviours for this group. This insight suggests that the dynamics of bullying behaviour differ between neurotypical and autistic adolescents, and highlight the need for the development of autistic-led assessment and support for bullying.

There is a growing literature on the incidence and perpetration of bullying in autistic adolescents which demonstrates that autistic adolescents are more likely than non-autistic peers to be involved in bullying both as perpetrators and victims (e.g., Abregú-Crespo et al., 2024; Ball & Zhu, 2023; Park et al., 2020). As such, it is important to understand if the function of bullying for autistic adolescents is the same as their neurotypical peers. Neurotypical adolescents’ goals in social interactions (e.g., status and dominance goals or prosocial communal goals) are associated with the extent to which they engage in bullying behaviour. However, to our knowledge, the social goals of autistic adolescents engaging in bullying behaviour have yet to be examined. An improved understanding of why autistic adolescents engage in bullying will facilitate the development of appropriate supports for autistic adolescents who bully others.

In the current study we examine the association between bullying behaviour and social goals across autistic and neurotypical adolescents. Notably, the autistic adolescents in this study attend a school that caters only to autistic young people, and as such provides a unique context for understanding the link between social goals and bullying behaviours in autistic individuals when engaging in within-neurotype social interactions. This will facilitate the development of relevant and targeted bullying prevention strategies for autistic adolescents, rather than relying on existing strategies framed within ‘normative’ social standards that position autistic social interaction as deficient, rather than simply different (see Davis & Crompton, 2021).

## Social Interactions and Bullying Behaviour

The incidence of victimisation in autistic adolescents is considerably higher compared to their non-autistic peers (e.g., between 46 and 94%; Adams et al., 2014; Park et al., 2020), however despite being frequent victims of bullying, research has also shown that autistic adolescents bully others to the same extent as their non-autistic peers (e.g., Ball & Zhu, 2023; Begeer et al., 2015; Rieffe et al., 2012; Van Roekel et al., 2010). Differences in the social insights and preferences of autistic adolescents suggests their bullying experiences may be different from their non-autistic peers. Indeed, qualitative research highlights differences in the experience of victimisation for autistic adolescents (e.g., Fisher & Taylor, 2016; Tipton-Fisler et al., 2018). Nonetheless, capturing peer- and self-reported bullying involvement is a common approach to assess autistic people’s experiences of bullying, as it is for neurotypical adolescents (Rieffe et al., 2012), and it is this approach that is taken in the current study.

Studies that have used both self- and peer-reported bullying behaviour questionnaires with autistic adolescents have found high levels of concordance across informants. For example, Van Roekel and colleagues (2010) found significant associations across self and peer reported bullying for 230 autistic adolescents, with an overall prevalence that corresponds to other studies also conducted in specialist provision, as well as accurate perceptions of bullying in film vignettes compared to neurotypical participants (although accuracy did differ as a function of autistic adolescents’ theory of mind). Similar concordance was found between peer- and self-reported bullying in a subset of the current sample (Begeer et al., 2015). Furthermore, a recent review on the assessment of bullying in autistic people highlights the importance of collecting information about bullying from multiple respondents (Morton, 2021).

Despite the growing literature on autistic adolescents’ bullying there is limited research examining the social function of bullying for autistic people. Autistic people often employ different social communicative skills from non-autistic people, which influences how they develop, maintain, and understand social relationships (Lord et al., 2020). As such, autistic adolescents’ social goals within bullying interactions may not be the same as their non-autistic peers. Furthermore, understanding within-neurotype interactions between autistic adolescents provides an important perspective in understanding why autistic adolescents engage in bullying behaviours, particularly in light of the double empathy problem.

The double empathy problem suggests that breakdowns in social interactions are the result of mutual misunderstandings between different neurotypes, rather than being the ‘fault’ of the autistic person’s differences in social interaction style (Milton, 2012). An emerging body of research has focused on understanding autistic people’s experiences with cross- versus same-neurotype interactions from a neurodiversity paradigm perspective, which challenges the view of autistic traits being deficits and instead considers them to be differences (e.g., Walker, 2021). Findings from this research (e.g., Crompton et al., 2020a, 2020b) suggest that autistic people often have more positive experiences when interacting with other autistic people compared to interactions with non-autistic people.

It might seem counterintuitive to explore bullying behaviours between autistic adolescents given the aforementioned research demonstrating that autistic people find interactions with other autistic people to be easier. However, like any heterogeneous group, autistic people experience within-group communication breakdowns as well (Milton et al., 2022). Rather than assuming that autistic people will not experience social difficulties when interacting with other autistic people, specific support should be developed based on autistic social preferences and norms rather than non-autistic social standards.

## Bullying and Interpersonal Goals

Previous research has found that neurotypical adolescents are often motivated to bully others to achieve status and dominance within their peer group (Olthof et al., 2011; Reijntjes, et al., 2013a; Sijtsema et al., 2009). Indeed, for neurotypical adolescents, bullying appears to serve its function as those who bully others are often perceived as popular and powerful by peers (Vaillancourt et al., 2003). Social goals within interpersonal interactions are often assessed using a self-reported questionnaire tapping different interpersonal outcomes within peer relationships (e.g., “you get to decide what to play”, “you can put others in a good mood”), such as the Interpersonal Goal Index (IGI-C; Ojanen et al., 2005). Findings from studies using the IGI-C in neurotypical adolescents have shown that social goals in peer interactions are consistently associated with peer reports of social behaviours (Ojanen et al., 2005; Sijtsema et al., 2009).

Two social goals that are frequently studied in relation to bullying behaviours are agentic goals (i.e., striving for dominance and leadership) and communal goals (i.e., striving for positive relationships with others). As expected, across a range of studies with neurotypical children and adolescents, aggressive and bullying behaviours were consistently positively associated with agentic goals and negatively associated with communal goals (Caravita & Cillessen, 2012; Goagoses et al., 2022; Pan et al., 2020; Samson et al., 2012; Sijtsema et al., 2009). However, the association between social goals and bullying behaviour is yet to be examined in autistic adolescents.

## The Current Study

The current study explores the association between adolescents’ identification with agentic and communal interpersonal goals and their involvement in bullying in two groups: (i) autistic adolescents attending specialist education provision, and (ii) neurotypical adolescents attending mainstream school. Given the unique setting of the current study (i.e., all autistic adolescents attended a school that solely catered to autistic students), we expect that the pattern of associations between social goals and bullying for autistic adolescents will be distinct from that seen in neurotypical adolescents. That is, the typical link between agentic goals and bullying behaviour will not be present for the autistic adolescents.

## Method

### Participants

Participants comprised two groups, both recruited from the broad vicinity of Amsterdam, the Netherlands. First, 108 autistic adolescents (99 boys (92%), *M*_age_ = 15.25 years, *SD* = 1.65 years; range: 11 to 18 years) were recruited from a single specialist education provision for autistic students preparing for tertiary education. To be admitted to this school, students were required to have been diagnosed with autism based on an assessment by a psychologist/psychiatrist and have average or above average IQ, and have perceived low support needs. Participants and their parents were informed about this study through presentations at school and letters sent home. Informed consent was obtained from all adolescents and their parents.

Second, 592 neurotypical adolescents (281 boys (47%), *M*_*age*_ = 13 years, *SD* = 0.5; range 11.1– 14.9 years) were recruited from five mainstream secondary schools. All participants were in their first year of secondary school. Opt-out passive consent was obtained, such that parents received information about the study and returned a letter to the school only if they did *not* want their child to participate, resulting in a 95% participation rate. Only 545 neurotypical adolescents completed both subscales of the IGI-C, and there was no significant difference between those that did and did not complete the questionnaire as a function of age or bullying behaviour, *t*s (590) < 1.51, *p*s > 0.132.

All procedures for this study were approved by the senior author’s university ethics board.

## Measures

### Interpersonal Goals

Interpersonal goals in social situations were assessed using the child version of the Interpersonal Goal Inventory which was designed for use with adolescents (IGI-C, Ojanen et al., 2005). The agentic and separate goals (3 items; e.g., “How important for you is it that you get to decide what to play”) and communal goals (4 items; e.g., “How important for you is it that you feel close to one others”) subscales were used in the current study. Participants indicated the extent to which they endorsed each item from, 0 = *not important to me at all,* to 3 = *very important to me* (Ojanen et al., 2005). The full set of items is presented in Table 1. Scores for each subscale were summed to create a total score (see Table 1). Internal consistency for the total sample was acceptable for agentic goals, Cronbach α = 0.77, and good for communal goals, Cronbach’s α = 0.81. Differences in the psychometric properties of the IGI-C across autistic and neurotypical adolescents are presented in the results.

**Table 1:** Percentage endorsing each item (rounded to nearest whole percent) (0 = *not important to me at all,* 1 = *not really that important to me*, 2 = *pretty important to me*, to 3 = *very important to me), Mean* (SD), Mean difference (*t*) for IGI-C items across autistic and neurotypical groups, and index of differential item functioning (L-A-LOR)

	Autistic					Neurotypical							
	*% 0*	*% 1*	*% 2*	*%n 3*	*M* (*SD*)	*% 0*	*% 1*	*% 2*	*% 3*	*M* (*SD*)	Mean difference (*t*)	Cohen’s *d*	DIF
Agentic goals													
Others agree to do what you suggest	7	17	48	29	1.99 (.85)	22	55	21	3	1.05 (.74)	− 11.72**	1.24	− 4.96**
You get to decide what to play	22	43	27	8	1.22 (.88)	26	56	16	3	0.96 (.72)	− 2.93**	0.35	5.12**
The group does what you say	17	27	36	21	1.60 (1.00)	27	53	18	2	0.95 (.73)	− 6.34**	0.82	0.40
Total	–				4.81 (2.00)	–				2.97 (1.86)	− 9.29**	0.95	–
Communal goals													
You feel close to the others	22	43	20	16	1.30 (.98)	3	9	57	31	2.15 (.72)	8.56**	1.12	− 3.03**
Everyone feels good	15	71	11	3	1.02 (.61)	3	12	57	29	2.12 (.71)	16.61**	1.60	0.21
You can put the others in a good mood	24	64	10	2	0.90 (.64)	3	16	67	14	1.92 (.64)	15.07**	1.59	0.70
Real friendship develops between you	39	48	11	2	0.76 (.73)	3	16	46	35	2.13 (.78)	16.76**	1.77	2.25*
Total	–				3.97 (2.09)	–				8.33 (2.10)	19.65**	2.08	–

^*^ *p* < 0.05, ** *p* < 0.01

DIF = Differential Item Functioning (Lor Z)

### Peer-Reported Bullying Involvement

Autistic and neurotypical adolescents reported on their peers’ bullying using the Bullying Role Nomination Procedure (BRNP) using the method outlined by Olthof et al. (2011) (see also Salmivalli et al., 1996). Importantly BRNP-based measures have shown stability over time (Reijntjes et al., 2013b) and associations with self-report measures of bullying (Bouman et al., 2012). Prior to completing this measure, participants were made familiar with a science-based definition of bullying comprising the features of intentionality, repetition, and power differential (see Olthof et al., 2011). They were then given descriptions of five different forms of bullying (i.e., physical, possession-directed (e.g., damaging property), verbal, direct relational and indirect relational) and after each description, asked to nominate classmates who carried out that form of bullying (see Olthof et al., 2011). Scores were computed for bully nominations by dividing the number of received nominations by the number of classmates who served as nominators. As adolescents typically engage in a particular type of bullying (e.g., physical or verbal bullying), instead of computing an overall mean across bullying behaviour, which likely underestimates the extent to which an individual engages in bullying, scores on the two highest forms of bullying were averaged (Spearman’s Rho = 0.89, *p* < 0.001) and used as their overall peer-reported bullying score (see Olthof et al., 2011; Witvliet et al., 2010). Final continuous scores showed severe kurtosis as, predictably, a large number of students were not frequently nominated by their classmates as engaging in bullying behaviours. As such, scores were transformed with a Rankit procedure (Soloman & Sawilowsky, 2009) resulting in an approximate normal distribution without outliers.

### Self-Reported Bullying

Following the peer-reported bully items, all participants rated the degree to which they bullied others by answering the question, “How often do you bully a classmate or participate in bullying a classmate yourself?” on a 5-point Likert scale, 1 = *almost never*, 2 = *rarely*, 3 = *sometimes*, 4 = *often*, and 5 = *very often* (Oldenburg et al., 2015)*.*

### Procedure

All data were collected by inviting participants belonging to a single class to complete a computerised version of the questionnaires either in their own classroom or in their school library. A trained research assistant gave group-wise instructions, emphasising the confidentiality of the study. To limit potential peer-influences on students’ responses, each participant completed the questionnaire in such a way that their peers were not able to see their responses.

### Analytic Strategy

Results are presented in two parts. Given the IGI-C has not previously been used with autistic adolescents, we compared the psychometric properties of the IGI-C across autistic and neurotypical groups prior to examining the association between social goals and bullying behaviour. First, the properties of the IGI-C for autistic compared to neurotypical adolescents are examined by testing the differential item functioning (DIF) of this measure across the two groups. In a DIF analysis, differences in the probability of endorsing an individual item given an overall score on a (sub)scale are compared across the target (autistic adolescent) and reference (neurotypical adolescent) groups. When individuals with the same overall score on a subscale have significantly different probabilities of endorsing an individual item, this suggests that the item behaves differently across the two groups.

The statistical approach taken to examine DIF was the standardised Liu-Agresti Common logs ratio (LOR-Z; Liu & Agresti, 1996) estimated using DIFAS 5.0 software (Penfield, 2005). Positive DIF values indicate that the item is more difficult for the target group (i.e., autistic participants) to endorse, and negative values indicate that the item is easier to endorse for the target group, given the same overall level of the underlying construct.

Second, the association between interpersonal goals and bullying for autistic and neurotypical adolescents was explored using a hierarchical regression model predicting bullying behaviour with the inclusion of interaction terms (i.e., group by interpersonal goal).

## Results

Previous studies (e.g., Bouman et al., 2012) have found robust associations between peer-reported and self-reported bullying. In the current study there was a significant positive association between self- and peer-reported bullying, total sample, *r*(701) = 0.29, *p* < 0.001, autistic adolescents *r*(107) = 0.35, *p* < 0.001, neurotypical adolescents *r*(593) = 0.28, *p* < 0.001. A total, multi-informant, continuous bullying score was therefore created by standardising and summing peer-reported and self-reported bullying scores.

### Properties of the IGI-C Across Autistic and Neurotypical Groups

Comparing the internal consistency of the IGI-C for autistic and neurotypical groups (Diedenhofen & Musch, 2016) revealed a significant difference for agentic goals, autistic group Cronbach’s α = 0.56, neurotypical group Cronbach’s α = 0.80; χ^2^(1) = 16.53, *p* < 0.001, but no such difference for communal goals, autistic group Cronbach’s α = 0.63, neurotypical group Cronbach’s α = 0.72; χ^2^(1) = 2.24, *p* = 0.134. DIF analysis also demonstrated differences across the two groups, with a large DIF value for several of the IGI-C items across both the agentic and communal subscales (see Table 1). Specifically, for the agentic scale, autistic adolescents were more likely to endorse, ‘others agree to do what you suggest’ and less likely to endorse, ‘you get to decide what to play’ compared to neurotypical adolescents with similar total agentic goal scores. For communal goals, autistic adolescents were more likely to endorse, ‘you feel close to others’ and less likely to endorse, ‘real friendship develops between you’, compared to neurotypical adolescents with a similar overall total communal goal score. Examining differences in the means of IGI-C items and subscale scores shows that autistic adolescents were more likely to endorse agentic goals, and neurotypical adolescents were more likely to endorse communal goals.

### Associations Between Interpersonal Goals and Bullying

Separate bivariate correlations for autistic and neurotypical adolescents (Table 2) show that agentic goals were positively associated and communal goals negatively associated with bullying for neurotypical adolescents. Neither agentic nor communal goals were associated with bullying for autistic adolescents. Notably, agentic and communal goals were significantly positively correlated for autistic adolescents only.

**Table 2:** Descriptive statistics by group and bivariate correlations for study variables (neurotypical above the diagonal, autistic below the diagonal)

	Autistic				Neurotypical								
	*n*	*M* (*SD*)	Range	Skewness	*n*	*M* (*SD*)	Range	Skewness	1	2	3	4	5
1. Sex	108	_	_	_	592	_	_	_	-	− .09*	− .22**	.27**	− .24**
2. Age (months)	108	183.01 (19.80)	137–213	− 0.35	592	155.77 (5.72)	133—179	0.14	.11	–	− .04	− .04	− .06
3. Agentic goals	107	4.81 (2.00)	0–9	−0.08	547	2.97 (1.86)	0—9	0.41	.01	− .19*	–	− .03	.19**
4. Communal goals	107	3.97 (2.09)	0–9	0.00	545	8.33 (2.10)	0—12	− 0.95	− .08	− .00	.51**	–	− .11*
5. Bullying	108	0.23 (1.92)	− 2.08–5.47	0.88	592	− 0.04 (1.54)	− 3.01 – 5.83	0.89	− .02	− .02	− .02	.16	–

^*^ *p* < 0.05, ** *p* < 0.01

A hierarchical multiple regression model was constructed to predict bullying. Sex and age were entered at the first step, followed by group (i.e., autistic vs. neurotypical) and interpersonal goals. Finally, interactions between group and interpersonal goals were examined on the third step (see Table 3). Variance inflation factors (VIF) were inspected to ensure that multicollinearity was not problematic. Across the three regression models, all VIF were below 2.90 (prior to the inclusion of the interaction terms at Step 3) suggesting that there were low levels of collinearity amongst the independent variables (Fox, 2015).

**Table 3:** Hierarchical multiple regression analyses predicting bullying

	Step 1		Step 2		Step 3	
	∆R^2^	β	∆R^2^	β	∆R^2^	β
	.05**		.01*		.01*	
Age		.01		.03		.01
Sex		− .22**		− .19**		− .17**
Group: autistic vs. NT		–		.08		.20
Agentic		–		.12**		.15**
Communal		–		− .03		− .07
Group*Agentic		–		–		− .15*
Group*Communal		–		–		.23*
Total *R*^*2*^	.05**		.06**		.07**	

NT = neurotypical

^*^ *p* < .05, ** *p* < .01

At Step 1, Sex made a significant independent contribution to the prediction of bullying, such that boys were more likely to engage in bullying compared to girls, *F*(2, 649) = 16.33, *p* < 0.001. The inclusion of group and interpersonal goals improved model fit, *p* = 0.039, and the overall model was still significant, *F*(5, 646) = 8.27, *p* < 0.001. Both sex and agentic goals were significant independent predictors at Step 2, such that higher agentic goal identification and boys compared to girls were both independently associated with greater bullying. At Step 3, the inclusion of the interaction terms further improved model fit, *p* = 0.013, and the overall model was significant, *F*(7, 644) = 7.21, *p* < 0.001. At this final step, in addition to sex and agentic goals, both interaction terms were significant.

To unpack these interaction effects, two further regression models were run to examine the role of agentic and communal roles for bullying behaviour separately for neurotypical and autistic adolescents. For neurotypical participants, only agentic, β = 0.14*, p* < 0.001, not communal goals, β = −0.05*, p* = 0.235, were a significant predictor of bullying behaviour. Conversely, for autistic participants only communal, β = 0.23*, p* = 0.043, not agentic goals, β = −0.14*, p* = 0.217, were predictive of bullying.

Given the relatively small number of girls in the autistic group, all analyses were re-run excluding both autistic and neurotypical female adolescents from the models. Notably, results remained unchanged, with the exception of the main effect for agentic goals which was no longer a significant independent predictor of bullying behaviour, although both interpersonal goal by group interaction effects remained significant.

## Discussion

This study set out to examine the association between interpersonal goals and bullying involvement in autistic adolescents, the majority of whom were male. Given this was the first study, to our knowledge, in which autistic adolescents completed the IGI-C, we first examined the measurement properties of this scale, finding differences across autistic and neurotypical groups. Second, differential associations between interpersonal goals and bullying were found across the groups. Importantly, autistic adolescents who reported greater communal goals also reported greater bullying, while for neurotypical adolescents there was an agentic-bullying link, as expected given the extant literature (e.g., Sijtsema et al., 2009). We discuss these findings in turn below.

There were several differences in autistic adolescents’ interpersonal goal identification compared to that of neurotypical adolescents. First, although neurotypical adolescents were more likely to endorse communal goals, autistic adolescents more readily identified with agentic goals, a pattern in line with autistic characteristics that may be interpreted as focusing greater attention on personal interests, leaving fewer resources for social interaction (Murray, 2018). Second, for autistic individuals, agentic and communal goal identification were significantly positively correlated; thus, despite a greater likelihood of endorsement of agentic goals, identification with these goals was not completely differentiated from identification with other social goals. Third, DIF analysis showed that there was variation across the two groups in the way individual items of the IGI-C contributed to both agentic and communal goal scores. Taken together, these findings suggest that the manner in which autistic adolescents identify with different social goals and apply them to their own self-concept diverges from that of neurotypical adolescents.

Broadly, these findings highlight the importance of examining the measurement properties of questionnaires when applying measures to different groups – particularly those groups for whom the questionnaire was not developed. While it was not an aim of this study to understand the implications of these findings for cross-neurotype interactions, the differences between autistic and non-autistic adolescents’ interpersonal goals in this context further underline the potential for social communication breakdown in line with the double empathy problem (Milton, 2012). Though, in this study, autistic adolescents are conceptualised as having diverged from the behaviour of neurotypical adolescents, it is important to remember that neither of these groups’ behaviour should be considered the ‘right’ or ‘wrong’ way of being, but rather examples of several different styles of social interaction.

Examining the association between interpersonal goals and bullying, we found, surprisingly, that autistic participants who reported identifying with communal goals engaged in greater bullying behaviour. This finding may highlight a relevant dynamic in the autistic group: communal goals imply greater motivation to engage in social interaction; however, they also make autistic adolescents more vulnerable to social misinterpretations. For instance, if an autistic adolescent fails to acknowledge another’s perspectives during an activity and pushes for their own desired course of affairs, they may be considered a bully, although they had the opposite intention. This finding complements recent work highlighting that autistic adolescents have difficulty classifying observed social scenarios as bullying (Hodgins et al., 2020). This finding also reinforces Milton et al.’s (2022) reflection that while the double empathy problem would suggest that autistic people are likely to connect with other autistic people more easily than with non-autistic people, within-neurotype interaction will not always be harmonious, and misunderstandings still exist.

It is important to emphasise that previous studies have shown that autistic adolescents are as accurate at reporting their own bullying behaviours as neurotypical adolescents (Van Roekel et al., 2010). As such, instead of a misinterpretation of their own bullying behaviour, it is likely that these adolescents are engaging in unintentional bullying of their peers at odds with their interpersonal goals. Clearly, further research is needed to unpack the dynamics of bullying interactions in neurodivergent populations. This is particularly necessary given that if bullying behaviour is indeed unintentional, it calls into question whether this behaviour can be classified as bullying as traditionally construed (Olweus, 1999). A better understanding of within-neurotype social interactions, including interactions that might be classified as bullying, can help facilitate a shift towards seeing autistic social interactions as different rather than deficient (Davis & Crompton, 2021). This will require a re-conceptualisation of many terms commonly used to classify and describe social interaction to better reflect the heterogeneity of expected behaviours and associated intentions.

## Limitations and Future Directions

The current study has a number of strengths, namely the precise matching of methods and procedures to both autistic and neurotypical adolescents to minimise potential differences arising as a function of method and the inclusion of both peer- and self-perspective of bullying behaviour. However, as in all research, some limitations of this work should also be acknowledged. First, although for both groups age was unrelated to the outcome variables, the age range of the autistic group was markedly larger than that of the neurotypical group. Given age-related differences have been found for self-presentation in autistic children and adolescents (Scheeren, et al., 2016), a focused examination of potential differences in interpersonal goal identification for autistic young people is warranted.

Second, the autistic adolescents in this sample were specifically selected because they all attended a single specialist education provision for autistic students. This allowed us to examine bullying behaviours amongst autistic adolescents and is well-aligned with the growing research interest in within-neurotype interactions (e.g., Crompton et al., 2020b; Morrison et al., 2020). However, our findings may not generalise to interactions between autistic adolescents in other specialist settings, or between autistic and non-autistic adolescents within mainstream schools; indeed, in line with the double empathy problem, we would expect bullying interactions, and social interactions more generally, between autistic and non-autistic adolescents to differ from interactions between autistic adolescents. Additional research is needed to explore this assumption. Importantly, the differences between groups may have also partially arisen due to specific bullying policies, procedures and teacher attitudes to bullying in mainstream schools. Understanding how school-level differences in the prevention and management of bullying shape adolescents’ bullying behaviours and beliefs is also an important direction for future research.

Third, there were notable differences in the psychometric properties of the IGI-C measure across the autistic and neurotypical groups, with specific items of both the agentic and communal scale more likely to be endorsed by autistic adolescents, alongside a low internal consistency for the agentic scale. This can be contrasted with the agreement found across peer- and self-reports of bullying behaviour found in the current paper and seen elsewhere both with traditional bullying (Van Roekel et al., 2010) and cyberbullying (Hwang et al., 2018). Together these findings suggest that although autistic adolescents appear to share an understanding of bullying behaviour (which may have been supported by the definition of bullying provided), they were less consistent in the interpretation of agentic social goals. Clearly, more work is needed to better understand how autistic individuals interpret and complete questionnaires designed and validated for neurotypical samples. Importantly, we hope that these findings will prompt the development of tools to assess social experiences that are designed and validated for the neurodivergent community.

Finally, the gender balance of the current study (92% male/8% female) is not representative of the 20% of autistic individuals that report being female (Lord et al., 2020). This may be due to the fact that all autistic adolescents in the current study were attending a specialist education provision for adolescents, and females tend to be diagnosed later in life (Begeer et al., 2013; Leedham et al., 2019). It is notable, however, that when girls are removed from both autistic and neurotypical groups, the main effect of agentic goals for bullying was no longer significant (although the group by agentic goals interaction remained). This result suggests that there are gender specific findings at play, supporting previous research that demonstrates gender differences in both social goals (e.g., Ojanen et al., 2005) and bullying (e.g., Cook et al., 2010) in neurotypical adolescents. However, no research, to our knowledge, has yet examined gender specific features of bullying behaviour in autistic adolescents, a clear omission given the differences between female-typical and male-typical autism presentation (Hull et al., 2020). Furthermore, given we did not collect information about the demographic features of participants (e.g., socio-economic status or ethnicity), further research is needed to examine the association between social goals and bullying behaviour across a wide range of autistic adolescents, as well as wide range of bullying behaviours, incliding cyberbullying.

We hope that these findings support the development of autism-specific measures of social interactions and intentions which better reflect autistic social experiences. Additionally, the findings reported here should encourage teachers and other practitioners working with autistic adolescents to ensure that guidance around preventing bullying and supporting victims of bullying is reflective of autistic young people’s experiences and understandings of bullying, rather than on neurotypical ideas of bullying. The development of such guidance should be co-created with autistic young people given that they are the most accurate sources of information about their own lived experiences.

In sum, the current paper provides an initial exploration of the differences in the association between interpersonal goals and bullying in autistic and neurotypical adolescents and suggests that the dynamics of bullying adolescents in specialist provision is different to that of adolescents in mainstream school settings. The development of new tools to understand social interactions that are designed specifically for neurodivergent adolescents will no doubt further our understanding of the social motivations of bullying in different educational contexts serving diverse students and enable the creation of neuro-affirming support for autistic young people.

## Data Availability

Data that support the findings of the current study are available from the corresponding author upon reasonable request.
